# Hirsutidin Prevents Cisplatin-Evoked Renal Toxicity by Reducing Oxidative Stress/Inflammation and Restoring the Endogenous Enzymatic and Non-Enzymatic Level

**DOI:** 10.3390/biomedicines11030804

**Published:** 2023-03-06

**Authors:** Faisal Imam, Preeti Kothiyal, Samiyah Alshehri, Muhammad Afzal, Muzaffar Iqbal, Mohammad Rashid Khan, Abdulrazaq Ahmed Hattab Alanazi, Md. Khalid Anwer

**Affiliations:** 1 Department of Pharmacology and Toxicology, College of Pharmacy, King Saud University, P.O. Box 2457, Riyadh 11451, Saudi Arabia; 2 School of Pharmacy and Research, Dev Bhoomi Uttarakhand University, Navagaon, Maduwala, Dehradun 248007, Uttarakhand, India; 3 Department of Pharmacology, Himalayan Institute of Pharmacy and Research, Rajawala 248007, Uttarakhand, India; 4 Department of Pharmaceutical Chemistry, College of Pharmacy, King Saud University, P.O. Box 2457, Riyadh 11451, Saudi Arabia; 5 Department of Pharmaceutics, College of Pharmacy, Prince Sattam Bin Abdulaziz University, Al-Kharj 16273, Saudi Arabia

**Keywords:** cisplatin, hirsutidin, nephrotoxicity, nuclear factor-kB, oxidative stress

## Abstract

Recent research has shown that phytocomponents may be useful in the treatment of renal toxicity. This study was conducted to evaluate the renal disease hirsutidin in the paradigm of renal toxicity induced by cisplatin. Male Wistar rats were given cisplatin (3 mg/kg body weight/day, for 25 days, i.p.) to induce renal toxicity. Experimental rats were randomly allocated to four different groups: group I received saline, group II received cisplatin, group III received cisplatin + hirsutidin (10 mg/kg) and group IV (per se) received hirsutidin (10 m/kg) for 25 days. Various biochemical parameters were assessed, oxidative stress (superoxide dismutase (SOD), glutathione transferase (GSH), malonaldehyde (MDA) and catalase (CAT)), blood-chemistry parameters (blood urea nitrogen (BUN) and cholesterol), non-protein-nitrogenous components (uric acid, urea, and creatinine), and anti-inflammatory-tumor necrosis factor-α (TNF-α), interleukin-1β(IL-1β). IL-6 and nuclear factor-kB (NFκB) were evaluated and histopathology was conducted. Hirsutidin alleviated renal injury which was manifested by significantly diminished uric acid, urea, urine volume, creatinine, and BUN, compared to the cisplatin group. Hirsutidin restored the activities of several antioxidant enzyme parameters—MDA, CAT, GSH, and SOD. Additionally, there was a decline in the levels of inflammatory markers—TNF-α, IL-1β, IL-6, and NFκB—compared to the cisplatin group. The current research study shows that hirsutidin may act as a therapeutic agent for the treatment of nephrotoxicity induced by cisplatin.

## 1. Introduction

Nephrotoxicity is a condition when harmful substances and medications cause the kidneys to excrete less toxic metabolic waste [1] and it can be identified by nuclear chromatin condensation, tubular dilatation, vacuolization of tubular cells, and microvilli loss [2]. In cases of severe renal injury, tubular degradation may involve solely the loss of the epithelial cell brush edge. The main histopathological characteristics of nephrotoxicity are apoptosis and necrosis of tubular epithelial cells [3,4]. Drug accumulation causes renal proximal tubule cells to exhibit significant toxicity, which ultimately results in tissue damage, poor perfusion, and renal failure [5]. The primary clinical characteristics of chronic kidney disease (CKD) are the sequential loss of vital functionalities, which, finally, results in chronic renal failure and requirement of kidney dialysis or transplants to preserve quality of life [6]. Acute kidney disease (AKD) is linked to a high prevalence of CKD, and people whose CKD is made worse by AKD have a higher death risk, according to earlier research [7]. A previous study indicates that a significant factor in about 60% of AKD cases in healthcare settings is drug-induced nephrotoxicity [8]. Cisplatin is used as a chemotherapy drug in treating different cancers, including testicular, ovarian, bladder, and lung cancers [4,9,10]. It works by interfering with the DNA in cancer cells, which prevents them from dividing and spreading [4,11,12]. Cisplatin is often used in combination with other chemotherapy drugs with significant side effects, including kidney injury and hearing loss [13,14]. The principal dose-limiting effect of cisplatin is nephrotoxicity, even though its remarkable anticancer activity is coupled with multiple toxicities including ototoxicity, gastrotoxicity, myelosuppression, and allergic responses [15,16,17]. The predominant location of impairment in cisplatin nephrotoxicity is the proximal tubules [17,18]. In addition to eliminating endogenous and external wastes, including medications, the kidney also retains some of these compounds in the proximal tubular region [19]. During cisplatin therapy, renal tissue accumulates cisplatin more quickly than other tissues and organs. The accumulation of cisplatin in these cells can lead to several pathophysiological consequences that contribute to its nephrotoxicity [20,21,22]. Cisplatin quantity in renal proximal tubule epithelial cells was found to be approximately five times higher than in serum [23].

The impact of cisplatin on DNA synthesis and repair, which inhibits cell proliferation as a result, is recognized as a contributing component to cisplatin toxicity [22,24,25]. An important factor in the acute renal failure induced by cisplatin is mitochondrial abnormality [25,26]. The accession of cisplatin inside the mitochondria turns into the production of reactive oxygen species (ROS), which then triggers oxidative stress and nephrotoxicity as well as renal damage [25,27]. Recent research suggests that oxidative stress significantly contributes to the proximal tubule injury caused by cisplatin, increasing the oxidation of lipids, proteins, and nucleic acids while decreasing the activity of enzyme-based antioxidants such as superoxide dismutase (SOD), glutathione peroxidase (GSH), and catalase (CAT) [17,28,29]. Low renal perfusion ultimately dictates the fate of the renal tissue by being indicative of nephrotoxicity and the necrosis of the proximal tubule’s terminal part. Renal failure brought on by cisplatin is indicated by a decreased rate of glomerular filtration along with increased levels of blood urea nitrogen (BUN)and plasma creatinine [22,30,31]. It was previously reported that the tumor-suppressor protein p53 is induced by cisplatin and, through the connection between the receptor and tumour necrosis factor, this impacts apoptosis. Cisplatin can also induce inflammation within the kidney, which can contribute to nephrotoxicity. This occurs when cisplatin stimulates the production of pro-inflammatory cytokines and chemokines, which recruit immune cells to the site of injury and can lead to tissue damage [32]. Therefore, the protective effects of various chemical and natural compounds with antioxidant activity against cisplatin-induced nephrotoxicity have been investigated [16,17]. The potential negative effects of cisplatin can be mitigated by several anti-inflammatory and antioxidant agents [33].

Therapeutic data and diagnostic data indicate that oxidative stress in the kidneys is generated by cisplatin administration resulting in the damage of tubules [34]. The reactive species of oxygen and nitrogen (ROS and RNS) have been proven to change the structural and functional integrity of membranes during mitochondrial respiration [35,36]. Furthermore, the build-up of these types of proteins in lysosomes and kidneys was used to elucidate the underlying cisplatin-induced acute nephropathy mechanism [37]. Numerous factors, including oxidative stress, inflammation, apoptosis, and dysfunctionality of mitochondria are symptomatic of cisplatin-induced nephropathy. However, the exact cause of the dysfunction is not fully understood [7].

Polyphenols called anthocyanins, present in fruits, vegetables, and flowers, are responsible for their pigmentation. It has been demonstrated that the flavonoid pigment of anthocyanin is naturally occurring and possesses antioxidant properties [38]. ROS are thought to be the cause of a wide variety of disorders; hence, it is hypothesized that this capacity serves as a defense mechanism. Consuming a diet high in anthocyanin-rich foods has been linked to a reduced risk of cancer [39], diabetes [40], obesity [40], bacterial infection [41], neurotoxicity [42], cardiovascular disease [43] and eye disease [44].

A member of the anthocyanin family, hirsutidin is an O-methylated anthocyanidin. It can be found in callus cultures and *Catharanthus roseus* (Madagascar periwinkle), where it is the most prevalent component in the petals. The review of the available literature reveals that the biological activities of the hirsutidin-plant flavonol glycoside, particularly its kidney protective capabilities, have received relatively little attention from scientific studies. The objective of the current study was to demonstrate the role of hirsutidin in the protection of the kidney in cisplatin-induced rats.

## 2. Materials and Methods

### 2.1. Animals

Male Wistar rats with a weight of 180 ± 20 g (*n* = 6 per group) were kept under standard laboratory conditions, including a 12 h–12 h light–dark cycle, humidity level of 40–50%, and room temperature of 23–28 °C. The animals were provided with regular feed and water prior to conduction of the experiment. Rats should have assimilated over the course of seven days while adhering to accepted laboratory procedures. The rodent experimentation was given final approval by the Institutional Animal Ethics Committee (IAEC/TRS/PT/022/018).

### 2.2. Drugs and Chemicals

Cisplatin was procured from Sigma (St. Louis, MO, USA). Hirsutidin was collected as a gift sample from SRL, India.Interleukin-1β (IL-1β), interleukin-6 (IL-6), tumor necrosis factor-alpha (TNF-α), and nuclear factor-kB (NF-κB) were determined by rat enzyme-linked immune-sorbent assay (ELISA) kit (MyBioSource, St. Louis, MO, USA).

### 2.3. Experimental Design

This proposed investigation was carried out according to the previous studies [45], with slight modifications. Four groups were divided randomly with 6 rats in each group.

1. Group I (Control group)—each rat received saline as a vehicle (p.o.) for 25 days.
2. Group II (Cisplatin control)—received cisplatin (3 mg/kg body weight/day, i.p.).
3. Group III (Test group)—animals were treated with cisplatin+ daily treatment of hisutidin orally (by gavage) with the dosage of 10 mg/kg.
4. Group IV (Per se group)—received daily treatment of hirsutidin orally (by gavage) with a 10 mg/kg dose.

To test for nephrotoxicity in rodents, the rats received cisplatin (3 mg/kg body weight/day) in the solution of saline (0.9%) which was injected every five days (total of four injections) until 25 days [7,46].

### 2.4. Biochemical Parameters

#### 2.4.1. Kidney Tissue Homogenate and Preparation of Biological Samples

Using metabolic cages, a sample of urine from 24 h was collected on the 25th day. At the endpoint, blood was collected using the retro-orbital technique. Approximately 200 μL to 1 mL of blood was taken and the separation of serum was performed by centrifugation for the duration of 20 min at the speed of 1000× *g*. A sample of serum and urine was taken to assess the various biochemical markers. After the rats were sacrificed, their kidneys were taken out and cleansed using normal saline solution and 10% *w*/*v* homogenates of tissue were prepared with the buffer of Tris-HCl (pH 7.5) with the molarity of 0.1 M and centrifuged at the speed of 3000× *g* for a time period of 15 min [7] to obtain supernatant. The kidney homogenates were used for the determination of oxidative stress and inflammatory markers.

#### 2.4.2. Estimation of Uric Acid, Urea, Creatinine, BUN, and Cholesterol

Using commonly accessible diagnostic kits (Meril life diagnostic kit, Gujarat, India), the serum at the absorbance of 254 nm and urine at the absorbance of 340 nm was evaluated spectrophotometrically to determine the uric acid (IFU/URCFSR01/01), urea (IFU/UREFSR01/00), levels of creatinine (IFU/CREFSR03/01), blood urea nitrogen (IFU/UREFSR01/00) and cholesterol (IFU/CHOFSR01/00) [7].

#### 2.4.3. Estimation of Biomarkers of Oxidative Stress

GSH activity was determined by the method involving the oxidation of NADPH into NADP+ in the presence of oxidizedglutathione [47] and the results were represented in µmol/g of protein. SOD activity was estimated through the inhibition of the formation of autocatalyzed adrenochrome in the presence of tissue homogenate at 480 nm and SOD was expressed as U/g protein [46]. The activity of catalase was determined by measuring hydrogen peroxide (H2O2) decomposition at 254 nm in the presence of CAT using the method used by Aebi et al. [48] and was expressed as U/g protein. Malondialdehyde (MDA) levels were determined as an index of the extent of lipid peroxidation in kidney tissue using standard method [49] and the results were represented in nmol/g of protein.

#### 2.4.4. Estimation of Inflammatory Markers

The supernatant was collected and used to measure levels of inflammatory markers, i.e., TNF-α (MBS175904), IL-1β(MBS732184), and NF-κB (MBS453975). The levels of TNF-α, IL-1β, and NF-κB in kidney tissue homogenate were quantitated using an ELISA assay according to the manufacturer’s instructions (My BioSource, St. Louis, MO, USA) [50].

#### 2.4.5. Estimation of Histopathological Investigations

For microscopic examination, histopathologic samples were collected from the renal tubules of rats in various groups and fixed in 10% formalin for 24 h. After washing in xylene, tissue samples were embedded in paraffin for 24 h at 56° in a hot-air oven. Using a sledge microtome, paraffin beeswax epithelial slabs were prepared for sectioning at a four-micron width. For microscopic examination, epithelial sections were positioned on the glass slides, deparaffinized, and stained with eosin stain and hematoxylin [51,52].

### 2.5. Statistical Analysis

Graph Pad Prism software (San Diego, CA, USA) version 8.0 was used to perform the analysis statistically for the current investigation. Results of statistical analysis were abridged in terms of standard error mean (SEM). Data were analyzed using one-way analysis of variance (ANOVA)followed by Tukey’s post-hoc test and values at *p* < 0.05 were considered statistically significant.

## 3. Results

### 3.1. Effects of Hirsutidinon Uric Acid, Urea, Creatinine, Urine Volume, BUN, and Cholesterol

**Figure 1:**
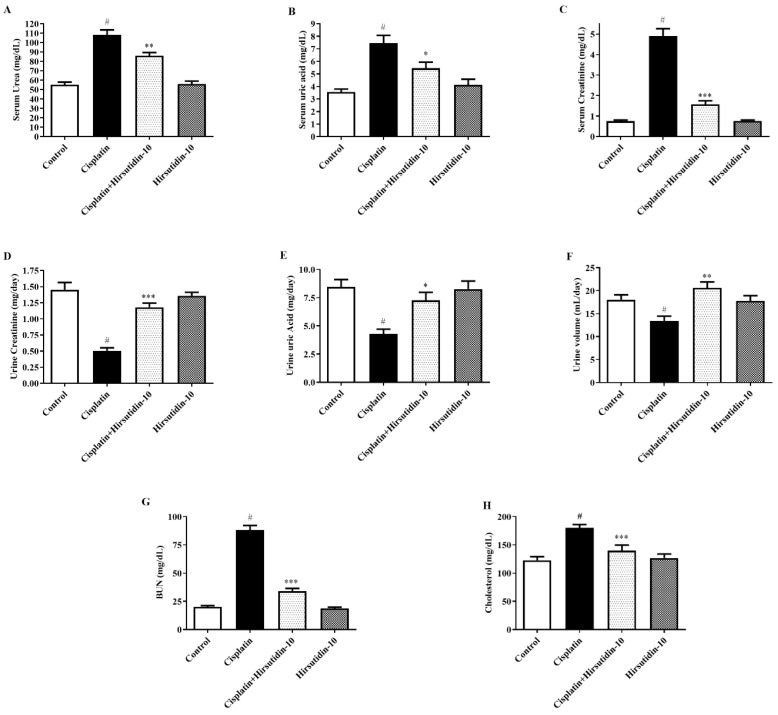
(**A**–**H**) Effect of hirsutidin on the evaluation of non-protein-nitrogenous components against nephrotoxicity induced in rats by cisplatin. (**A**) Serum urea. (**B**) Serum uric acid. (**C**) Serum creatinine. (**D**) Urine creatinine. (**E**) Urine uric acid. (**F**) Urine volume. (**G**) BUN. (**H**) Cholesterol. Mean ± SEM (n = 6). One-way ANOVA followed by Tukey’s post-hoc test. # *p* < 0.001 vs. control (group I), * *p* < 0.05, ** *p* < 0.01, *** *p* < 0.001 vs. cisplatin (group II).

Figure 1A–H shows the effect of hirsutidin on uric acid, urea, creatinine, urine volume, BUN, and cholesterol in Wistar with renal dysfunctionality induced by cisplatin administration. The serum levels of urea, uric acid, and creatinine in group II were remarkably higher compared to group I, indicating that group II experienced a notable change in the excretion of the aforementioned parameters. Similarly, the present study concurrently evaluated the parameters of urine such as creatinine, uric acid, and urine volume to identify the unsafe levels of the stated elements as an outcome of nephrotoxicity in rats induced by cisplatin. Group II showed a notable downregulation in urine creatinine, urine uric acid, and urine volume, with increased serum levels of urea, uric acid, and creatinine as compared with group I. Tukey’s post-hoc test revealed that group III significantly restored the raised level of urea in serum (F (3, 20) = 48.49, (*p* < 0.0001)), serum uric acid (F (3, 20) = 13.69, (*p* < 0.0001)), and serum creatinine level (F (3, 20) = 96.43, (*p* < 0.0001)) as compared to group II. Similarly, notable variations in group III were detected in the urine parameters such as creatine (F (3, 20) = 33.92, (*p* < 0.0001)), uric acid (F (3, 20) = 9.094, (*p* < 0.0001)), and urine volume (F (3, 20) = 7.118, (*p* < 0.0001)), as compared with group II. Group IV did not show any significant changes. We found that group II showed a substantial elevation in the levels of certain serum chemistry parameters such as BUN and cholesterol levels as compared to group I. Group III showed notable changes in the serum chemistry parameters with BUN (F (3, 20) = 167.2, (*p* < 0.0001)), and cholesterol (F (3, 20) = 12.39, (*p* < 0.0001)), in comparison to group II, as evidenced by Tukey’s post-hoc test. Group IV experienced no statistically significant changes compared to group I.

**Effects of hisutidin on oxidative-stress parameters**

**Figure 2:**
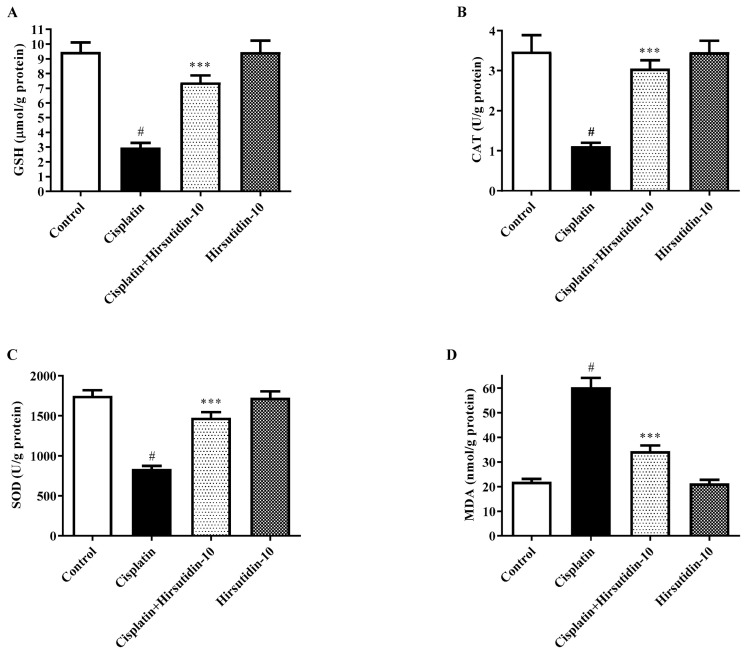
(**A**–**D**) Effect of hirsutidin on the parameters of oxidative stress against nephrotoxicity induced by cisplatin in rats. (**A**) GSH. (**B**) CAT. (**C**) SOD. (**D**) MDA; one-way ANOVA followed by Tukey’s post-hoc test. # *p* < 0.001 vs. control (group I), *** *p* < 0.001 vs. cisplatin (group II).

Hirsutidin effects on rat levels of non-enzymatic and enzymatic parameters against nephrotoxicity induced by cisplatin administration are depicted in Figure 2A–D. In the current analysis, group II exhibited substantial changes in several enzymatic activities along with levels of a range of non-enzymatic oxidative-stress biomarkers, which are linked to nephrotoxic events in the kidney tissues. Group II rats showed a remarkable decline in enzymatic activities, such as CAT and GSH, as well as the nonenzymatic biomarker level of SOD, which is regarded as a key biomarker for ROS, in the tissues of a kidney when compared with group-I rats. In addition, group II showed significantly increased levels of MDA, a crucial oxidative biomarker, in kidney tissue (*p* < 0.05). Tukey’s post-hoc test indicated that group III significantly restored the activities of several antioxidant enzymes parameters as compared to group II i.e., CAT (F (3, 20) = 16.73, (*p* < 0.0001)), GSH (F (3, 20) = 27.72, (*p* < 0.0001))and SOD(F (3, 20) = 41.96, (*p* < 0.0001))along with the normalized levels of oxidative stress indicators MDA (F (3, 20) = 57.31, (*p* < 0.0001)), which shows the significant variations related with nephrotoxicity induced in the models of animal for experimentations. The rats did not show significant changes in group IV compared to group I.

### 3.2. Effects of Hrisutidin on Inflammatory Markers

**Figure 3:**
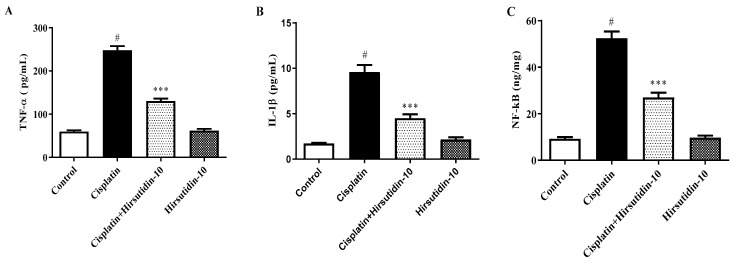
(**A**–**C**) Effects of hirsutidin on anti-inflammatory parameters for the nephrotoxicity induced by cisplatin in rats. (**A**) TNF α. (**B**) IL-1β. (**C**) NF-kB; one-way ANOVA followed by Tukey’s post-hoc test. # *p* < 0.001 vs. control (group I), *** *p* < 0.001 vs. cisplatin (group II).

Figure 3A–C depict the impact of hirsutidin on anti-inflammatory markers in rats against the nephrotoxicity induced by cisplatin administration. Group II exhibited noticeably elevated tissue pro-inflammatory levels of cytokines, i.e., TNF-α, IL-1β, and NF-kB. Tukey’s post-hoc test revealed that group III rats showed a significant decline in the levels of inflammatory markers, i.e., TNF-α (F (3, 20) = 218.5, (*p* < 0.0001)), IL-1β (F (3, 20) = 59.06, (*p* < 0.0001)), and NF-kB (F (3, 20) = 119.9, (*p* < 0.0001)) in comparison to the group-II rats. Furthermore, group IV did not show any significant changes compared to group I.

### 3.3. Effects of Hirsutidin on Histopathological Investigations

**Figure 4:**
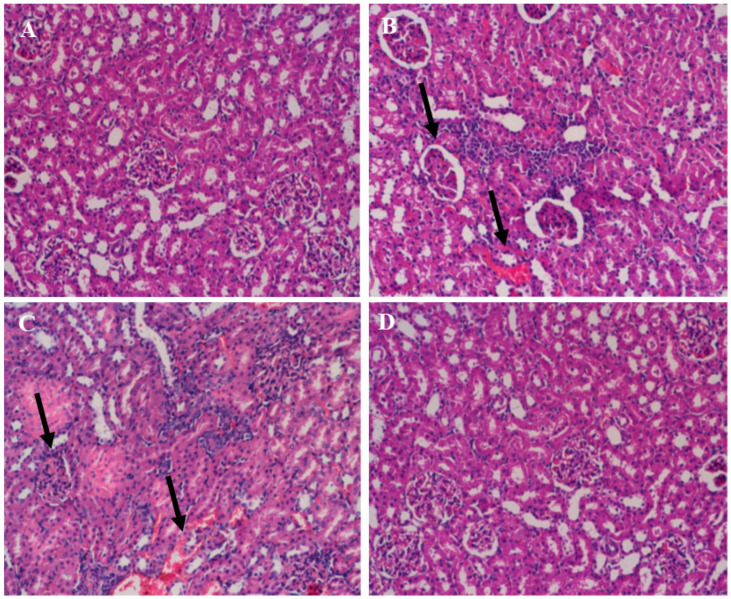
(**A**–**D**) Photomicrograph of rat kidney tissue section. (**A**) Normal histology of the kidney tissue in group I (control) shows the normal architecture of the glomerulus and tubules (H&E × 200). (**B**) Group II (Cisplatin) showing, with the black arrow, extensive degeneration, acute tubular necrosis, hyaline casts, interstitial edema, and inflammatory cell infiltration (H&E × 200). (**C**) Group III (test) showed improved histopathologic resembling group II (H&E × 200). (**D**) Group IV (per se) shows normal architecture (H&E × 200).

Figure 4A–D displays the histopathological modifications caused by the administration of cisplatin and hirsutidin to kidney tissues. Group I and group IV showed that the glomerulus and tubules are normal with normal architecture. As a result, the kidneys of the group-II rats displayed extensive degeneration, acute tubular necrosis, hyaline casts, interstitial edema, and inflammatory cell infiltration with the disruption of normal architecture. In contrast, group-III rats showed less histopathologic damage than group I.

## 4. Discussion

Cisplatin is the most widely used drug in the treatment of different cancers and solid tumors. However, the main obstacle to the widespread clinical use of this drug as a long-term treatment is the fact that it causes nephrotoxicity [30,53,54]. The nephrotoxicity caused by cisplatin is well-known to be caused, in part, by oxidative stress and inflammation, both of which play a significant role in the development of nephrotoxicity [55,56,57]. This study was conducted to reveal whether hirsutidin can be used to prevent the development of kidney damage by cisplatin-induced nephrotoxicity.

ROS are produced as a result of the inhibition of anticancer medication for the respiratory complex of mitochondria in renal tubular cells, which leads to tissue and organ damage [22,58,59]. Lipid peroxidation, as well as modifications to the enzymatic and nonenzymatic antioxidant systems along with alterations in gene expression, have been brought on by oxidative stress arising in renal tissue and the production of ROS [30,60]. It is considered that oxidative stress and inflammation are linked to abnormalities in the structure and functioning of renal tissue. Chemokines such as TNF-α were responsible for causing inflammation. Apoptosis is involved in nephrotoxicity caused due to cisplatin administration, as evidenced by the previous literature [61,62].

Past research has shown that cisplatin is recognized by most people to induce acute renal impairment [30]. This is the cellular process underlying kidney injury etiology [7,63]. Additionally, clinical concepts are advanced that contend that any structural and functional anomalies found in renal tissues are related to the emergence of inflammatory and oxidative stress. The current study looked at the potential nephroprotective effectiveness of hirsutidin against cisplatin-induced renal damage in rats. Cisplatin-induced neurotoxicity has been associated with damage to mitochondria and nuclei. In the current study, the experimental animal paradigm was used to examine the nephrotoxicity caused by cisplatin. As per the results of a previous study, several biomarkers are produced during the pathology of chemically induced nephrotoxicity. The changed amounts in non-protein-nitrogenous substances such as serum levels of urea, uric acid, and creatinine in the blood were also hypothesized by prior evidence [60].

Additionally, it was discovered that the levels of creatine in urine, the volume of urine, and the level of urine uric acid in experimental animal models were altered [64]. In the current study, we considered the kidney homogenate’s nonprotein-nitrogenous component levels. In the 25-day exploratory procedure, the administration of cisplatin significantly changed the amounts of the aforementioned non-protein components, elaborating upon the harmful impact of cisplatin on physiological activities. On the contrary, rats treated with hirsutidin (10 mg/kg) restored levels of all the biological components.

As high cholesterol levels might contribute to the onset of kidney disease, evaluating cholesterol levels is crucial for determining renal (kidney) function [65,66]. The filtering of waste from the bloodstream and the control of many chemicals, including cholesterol, are important functions of the kidneys [67,68]. The risk of cardiovascular disease increases when the kidneys are not working correctly because high cholesterol levels can cause plaque to accumulate in the arteries [69,70]. Numerous evidence-based studies point to the importance of blood chemistry in clinical signs of nephrotoxicity induced chemically [45]. According to previous research, changes in kidney function are linked to considerable variations in the level of several blood nephrotoxicity indicators, including BUN and serum cholesterol [71]. In this investigation, we found that the serum level of BUN and serum cholesterol were significantly impacted by the injection of cisplatin. The sensitive filters in the kidneys can also be harmed by an excess of cholesterol, which over time will result in a reduction in renal function [72,73]. Examining cholesterol levels can also reveal additional risk factors for kidney disease such as diabetes, high blood pressure, and a family history of kidney issues [74,75]. In line with this, we found in our study that doses of hirsutidin over the course of a protocol favorably improved their blood-chemistry profiles, indicating that hirsutidin may be a product of the nephroprotective effect in cisplatin-induced kidney damage.

These biochemical indicators have also notably highlighted the importance of non-protein-nitrogenous component estimation, blood-chemistry analysis, along with the concentrations of significant non-enzymatic and enzymatic biomarkers. With a primary emphasis on antioxidant enzymes such as CAT, GSH, MDA, and SOD in the tissues of the kidney, earlier investigations clarified the relevance of oxidative parameters in the etiology of nephrotoxicity. Furthermore, earlier research demonstrated that the activity of GSH, CAT, and SOD in the kidney tissues significantly decrease in cisplatin-induced nephrotoxicity. When examining the kidney profiles, however, dramatically increased amounts of MDA were discovered. In this study, we revealed that the kidney homogenate of the rat group that ingested cisplatin showed remarkable changes in the activity of GSH, CAT, and SOD together with higher levels of MDA. Post-treatment with hirsutidin for 25 days showed the restoration of the entire activity along with the levels of biomarkers, which is an indication of hirsutidin’s protective effects in the rats against cisplatin-induced nephrotoxicity.

Inflammatory markers play a very crucial role in the pathogenesis of nephrotoxicity induced by cisplatin administration [52]. The effect of the number of chemokines and inflammatory cytokines is elevated in the kidney after cisplatin ingestion [45]. Nuclear transcription factor NF-kB is mainly triggered by the cytokines IL-1β and TNF-α. The reduced NF-kB levels in the following study can perhaps be explained by the theoretical means: that cisplatin tends to cause NF-kB inhibition by binding the regions of “kB” that are the binding regions of NF-kB [51]. The expression of COX-2 which is linked to the proliferation of cells is mediated by the activated NF-kB [76]. The levels of TNF-α, IL-1β, and NF-kB were upregulated in cisplatin-treated rats in the present study. TNF-α triggers the receptor-dependent cascade of apoptosis after binding to its receptors through the activation of caspase-3. An administration of hirsutidin decreased the expression of TNF-α, IL-1β, and NF-kB. Hirsutidin improved renal histology with only mild swelling and vacuolations of epithelial lining renal tubules. The administration of hirsutidin revealed a relative improvement in the condition of histopathological damage induced by cisplatin [77,78]. In rats with warm renal injury, hirsutidin reduced acute tubular necrosis. Additionally, it reduced degeneration, hyaline casts, tubular necrosis, interstitial edema, and inflammatory cell infiltration in rats with contrast-induced nephropathy [78,79]. In our study, hirsutidin treatment significantly protected against nephrotoxicity caused by cisplatin treatment. Based on biochemical evidence and histological evidence, hirsutidin might be beneficial in reducing cisplatin toxicity due to its antioxidant and active ingredient properties. The present study showed that hirsutidin prevented cisplatin-induced kidney injury by modulating oxidative stress and marker enzymes. Furthermore, hirsutidin has been shown to reduce biochemical changes in the kidneys, such as increased levels of creatinine, urea, uric acid, and BUN. By reducing oxidative stress, hirsutidin helps to protect cells from damage caused by the harmful effects of ROS. Hirsutidin has been found to regulate the activity of marker enzymes involved in cellular metabolism, such as transaminases, inflammatory markers, and NF-kB, which can also play a role in preventing kidney injury. Long-term exposure to hirsutidin can provide a more comprehensive understanding of its effects and its mechanism of action. By conducting additional studies using techniques such as Western blotting, cell-based assays, and gene expression analyses, researchers can obtain a better idea of how hirsutidin impacts cellular processes and molecular mechanisms. Use of a small number of animals in the study is one of the limitations and makes it difficult to detect rare or indirect effects of hisutidin on the nephrological parameters.

## 5. Conclusions

The present study evaluated the data referring to the effect of hirsutidin’s potential nephroprotective activity, for the first time, to evaluate its ability to restore some biochemical indicators of oxidative stress with alter enzymatic and non-enzymatic components and change blood-chemistry profile information in cisplatin-induced neurotoxic effects. Additionally, hirsutidin might exhibit nephroprotective effects, as evidenced by its ameliorative efficacy in non-protein-nitrogenous components along with restoration of inflammatory markers altered as an outcome of induced nephrotoxicity. According to the aforementioned data, the natural supplement hirsutidin may have the potential to be an effective treatment for drug-related nephrotoxic side effects.

## Data Availability

All the data generated in this study have been included in the manuscript.
